# Involvement of propranolol in suicides: cross-sectional study using coroner-reported data

**DOI:** 10.1192/bjo.2024.714

**Published:** 2024-06-03

**Authors:** Hayley C. Gorton, Charlotte Archer, Thikra Algahtani, Faraz Mughal, Caroline S. Copeland

**Affiliations:** Aston Pharmacy School, Aston University, UK; and School of Applied Sciences, University of Huddersfield, UK; Bristol Medical School, University of Bristol, UK; Centre for Pharmaceutical Medicine Research, Institute of Pharmaceutical Science, King's College London, UK; and National Programme on Substance Use Mortality, London, UK; School of Medicine, Keele University, UK

**Keywords:** Suicide, primary care, epidemiology, antidepressants, propranolol

## Abstract

**Background:**

Propranolol is a beta-blocker medication indicated mostly for heart rhythm conditions and for physical symptoms of anxiety. Prescriptions for propranolol in the UK have increased since 2008. Recently, there have been concerns about the involvement of propranolol in intentional poisonings, but such deaths are not routinely reported. Therefore, use of coroner-reported and toxicology data enables unique investigation into the scale of involvement of propranolol in suicide.

**Aims:**

To describe the extent to which propranolol is involved in suicides, including patterns over time and characteristics of people whose suicide involved propranolol compared with other suicides.

**Method:**

Data were derived from the National Programme on Substance Use Mortality (NPSUM). All suicides and deaths of undetermined intent between 2010 and 2021 in England, Wales and Northern Ireland were extracted, and a subset was identified where propranolol was involved in death.

**Results:**

There were 4473 suicides of which 297 (6.6%) involved propranolol, with the proportion involving propranolol nearly quadrupling during the study period (3.4% *v*. 12.3%). Compared with all other suicides, a greater proportion of propranolol suicides were in women (56.6% *v*. 37.1%) and in people with diagnoses of depression (39.1% *v*. 27.1%) and anxiety (22.2% *v*. 8.6%). When suicide involved propranolol, an antidepressant was detected at post-mortem in 81.8% of deaths, most commonly a selective serotonin reuptake inhibitor (SSRIs) (51.5%), and most often citalopram (24.6%).

**Conclusions:**

A small number, but increasing proportion, of suicides reported to the NPSUM involve propranolol. Vigilance to the combined toxicity profile of medicines used alongside propranolol may be pertinent.

Propranolol is a beta-blocker medication indicated for various conditions, mostly related to heart rhythm.^1^ It is also licensed for the physiological symptoms of anxiety including sweating, tachycardia and tremor. It is used either as a regular or ‘as needed’ treatment, but it is not included in the National Institute for Health and Care Excellence guidelines for any anxiety disorder.^2^ According to open-access UK prescribing data, almost 600 000 prescriptions of propranolol are dispensed monthly.^3^ The prevalence of propranolol prescribed for patients with anxiety recorded in general practice more than doubled from 3.8/1000 person-years at risk in 2008 to 8.7/1000 person-years at risk in 2018, and the most substantial increase in prescribing was in young adults (age 18–35).^4^ Reasons for this rise could include: general practitioners (GPs) prescribing propranolol more readily than other medications as they describe propranolol as non-addictive;^5^ a reduction in incident benzodiazepine prescribing in alignment with national guidance;^4^ and GPs wanting to ‘do something’ while waiting for referral to psychological therapies, as has been reported for antidepressant prescribing in depression.^6^

Recently, concerns about the involvement of propranolol in poisoning have been described following investigation by the Healthcare Safety Investigation Branch (HSIB).^7^ The resulting safety recommendations included updates to national formularies and guidance related to toxicity of propranolol in overdose; a requirement for professional bodies to support doctors and pharmacists in identifying potential prescribing risks to at-risk groups; and guidance about how the ambulance service responds to and manages overdoses. In their 2022/2023 annual report, the National Poisons Information Service (NPIS) in the UK designated propranolol as an ‘area of interest’ and are in the process of making recommendations about prescribing appropriateness and risk of harm in overdose.^8^

In 2014, the Australian Therapeutic Goods Administration recommended caution in prescribing propranolol to people at risk of self-harm, or to prescribe small quantities, following coroner suggestions.^9^ In 2019, after a suicide poisoning with propranolol, a UK coroner issued a Regulation 28 Report to Prevent Future Deaths to the chair of the regulator for doctors and the Health Secretary of the UK government to encourage learning from the circumstances surrounding the death.^10^ The Advisory Council on the Misuse of Drugs have included propranolol on a ‘watch list’, presumably because of concerns about misuse although there are no published criteria for medicines on this list.^11^ In the UK, clinicians are encouraged to refer anyone who has self-harmed through ingestion of propranolol for psychiatric and medical assessment.^1^ The latest NPIS report advocates for consideration in appropriateness of prescribing of propranolol in people with a history of self-harm.^8^ Of the 82 deliberate overdose enquiries to two NPIS units in a one-year period, 27% had history of prior overdose.^12^

Between 1993 and 2017, propranolol was listed on 768 death certificates for drug-poisoning deaths in England and Wales.^13^ These data were obtained from a freedom of information request, and propranolol is not routinely reported in Office for National Statistics (ONS) drug-related death data. Therefore, little is known about the profiles of these individuals.

The aim of this study was to describe the extent to which propranolol is involved in suicides. The objectives were to: (a) describe the temporal pattern of propranolol involvement in suicides; (b) describe the characteristics of people whose death involved propranolol; and (c) compare these characteristics with other suicides.

## Method

The study is reported in accordance with the Strengthening the Reporting of Observational Studies in Epidemiology Statement guidelines^14^ (Supplementary Material 1 available at https://doi.org/10.1192/bjo.2024.714).

### Transparency declaration

All authors affirm that the manuscript is an honest, accurate and transparent account of the study being reported.

### Data sources

The National Programme on Substance Use Mortality (NPSUM, previously known as the National Programme on Substance Abuse Deaths (NPSAD)) receives information from coroners in England, Wales and Northern Ireland on a voluntary basis on deaths related to drug use. If a death has an unknown cause, is violent or unnatural, sudden and unexplained, occurred during an operation or before the person came out of an anaesthetic, or may have been caused by an industrial disease or poisoning, then it is referred to a coroner. These criteria therefore encompass all deaths suspected to have occurred by suicide. Toxicology tests are requested at the discretion of the coroner and/or pathologist, dependent upon individual circumstances. Coroners report a death to the NPSUM if one or more psychoactive substance(s) is detected at post-mortem by analytical toxicology testing and/or directly implicated in causing death at inquest, or if the decedent had a history of drug (mis)use. Coroners designate a substance as implicated if they believe that it contributed partly or wholly to the death. The NPSUM data fields include details of death including location, circumstances, coroner-reported conclusions, causes of death, narrative supplementary details and post-mortem toxicology detailing drugs detected. Demographic details about the person are reported, and this can include medical history. The King's College London Biomedical & Health Sciences, Dentistry, Medicine and Natural & Mathematical Sciences Research Ethics Sub-Committee re-confirmed (August 2023) that the NPSUM does not require ethics review as all subjects are deceased.

### Population

This was a cross-sectional study. All suicides occurring between 2010 and 2021 were extracted. The definition of suicides used by the ONS in England includes deaths caused by intentional self-harm in people aged ten and over and those of undetermined intent aged 15 and over.^15^ These are the definitions used in this study. The time period was selected because 2010 was the earliest year for which data about mental health diagnoses were complete, and 2021 is the latest year for which there is near-complete data entry into the NPSUM. The subset of suicides where propranolol was involved were extracted based on the presence of propranolol either at post-mortem and/or implicated in death. Where submissions are made to the NPSUM and data processors confirm eligibility, they do include propranolol due to its potential indication for the physiological symptoms of anxiety. Generally, coroners indiscriminately submit drug-related deaths for the NPSUM data processors to determine which are in the scope of the NPSUM. However, where coroners submit records based on their assessment of psychoactive drug involvement, it is possible that they would not describe propranolol as psychoactive. In these situations, deaths involving propranolol would only be reported if other psychoactive medications were concomitantly involved.

### Data cleaning

Data were cleaned to confirm validity by H.C.G., T.A. and C.S.C. This included cross-checking implicated drugs with cause of death and coroner conclusions. Where discrepancies existed, the original coroner-submitted documents were reviewed, and entries altered or confirmed as appropriate. Method of suicide was assigned to create a categorical variable based on method described in cause(s) of death.

### Data analysis

Data were managed and analysed using IBM SPSS Statistics (version 28). Descriptive statistics were undertaken. Comparisons were made between the subset of suicides where propranolol was involved and all suicides that were reported to the NPSUM, using statistical tests appropriate for the variable distributions. Microsoft Excel was used to produce graphs. Joinpoint Regression Program^16^ was used to examine temporal trends and identify years where statistically significant changes in trends occurred and produce associated figures.

## Results

### Overall summary

Between 2010 and 2021 there were 4473 suicides and deaths of undetermined intent reported by coroners from England, Wales and Northern Ireland to the NPSUM. In 297 (6.6%) of these deaths, propranolol was detected at post-mortem for all but one (in a case where no toxicology screening was done) and implicated in 184 deaths (62.0%). Propranolol was equally as likely (*P* = 0.694) to be implicated in death if it was prescribed (*n* = 66/104, 63.5%) compared with when it was not prescribed (*n* = 118/193, 61.1%). A total of 91% of suicides involving propranolol were medicines poisoning, 5% were hanging and the remainder were other methods.

**Fig. 1:**
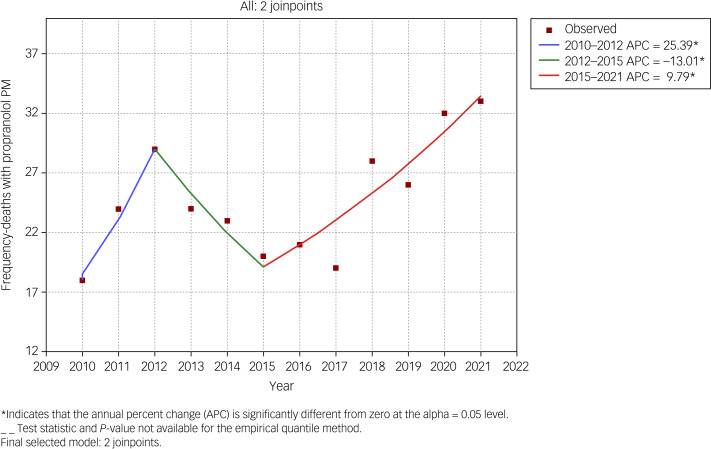
Join point plot of temporal trends in propranolol suicides between 2010 and 2021. PM, post-mortem.

**Fig. 2:**
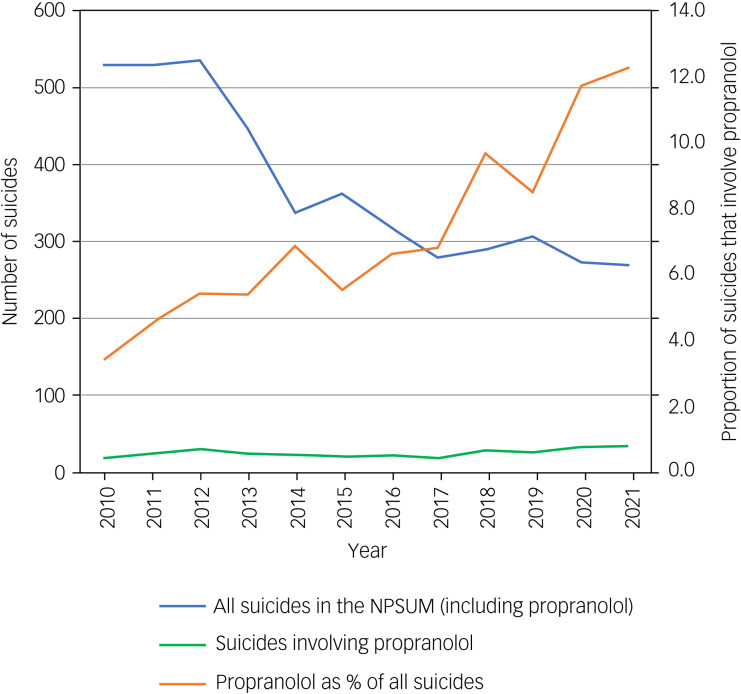
Line graph of the number of all suicides, those involving propranolol and the proportion of suicides involving propranolol between 2010 and 2021. NPSUM, National Programme on Substance Use Mortality.

There were between 18 (2010) and 33 (2021) suicides involving propranolol each year. When calculated with zero join points, a relatively constant trend across the whole time period was observed. Join point regression with two-join points showed statistically significant (*P* < 0.05) trends in three time periods (Fig. 1). There was an annual percentage increase in the number of reported suicides involving propranolol of 25.4% between 2010 and 2012, followed by a 13.0% decrease between 2013 and 2015, and 9.8% increase between 2015 and 2021. Suicides involving propranolol as a proportion of all suicides nearly quadrupled between 2010 (*n* = 18/529, 3.4%) and 2021 (*n* = 33/269, 12.3%), whereas numbers of suicides overall declined over time (Fig. 2).

### Demographics

Most suicides involving propranolol were in women (56.6%) compared with all other suicides which were more common in men (62.9%) (Table 1). The median age at death was 44 (interquartile range (IQR) 33–51) when propranolol was involved and 46 (IQR 35–56) when propranolol was not involved. The proportion of suicides involving propranolol represented by the age 65 and over age band (*n* = 12/297, 4%) was lower than the proportion of all suicides in this age band (*n* = 589/4176, 14.2%). The spread of occupational status was different in the propranolol cohort, who were more likely to be employed or students, and less likely to be retired than all other suicides. There was no difference in living arrangements between the groups. Across the whole data-set, ethnicity was recorded as white in 58% of deaths, with ethnicity not recorded for 40% of the deceased.

**Table 1:** Baseline demographics of subset where propranolol was involved and all other suicides

Demographic	Propranolol (*n* = 297)	All other suicides (*n* = 4176)
Female*	168 (56.6%)	1551 (37.1%)
Age band*		
≤14	0	2 (0%)
15–24	30 (10.1%)	319 (7.6%)
25–34	53 (17.8%)	671 (16.1%)
35–44	68 (22.9%)	955 (22.9%)
45–54	98 (33.2%)	1042 (24.9%)
55–64	36 (12.0%)	598 (14.3%)
≥65	12 (4.0%)	589 (14.2%)
Occupation status*		
Unknown	49 (16.5%)	531 (12.7%)
Unemployed	86 (29.0%)	1356 (32.5%)
Employed	116 (39.0%)	1298 (31.1%)
Homemaker	4 (1.3%)	87 (2.1%)
Student	11 (3.7%)	94 (2.3%)
Retired	18 (6.1%)	636 (15.2%)
Unable to work because of ill health	13 (4.4%)	150 (3.6%)
Other	0	24 (0.5%)
Living arrangements		
Unknown	61 (20.5%)	736 (17.6%)
No fixed abode	0	36 (0.9%)
Alone	123 (41.4%)	1811 (43.4%)
With others	111 (37.4%)	1545 (37.0%)
Other	2 (0.7%)	48 (1.1%)
Diagnoses		
Addiction*^a^	44 (14.8%)	1052 (25.2%)
Anxiety*	66 (22.2%)	360 (8.6%)
Schizophrenia*	1 (0.3%)	96 (2.3%)
Eating disorder	1 (0.3%)	26 (0.6%)
Depression*	116 (39.1%)	1133 (27.1%)
Bipolar disorder	7 (2.4%)	110 (2.6%)
Post-traumatic stress disorder	3 (1.0%)	21 (0.5%)
Known mental health condition^b^	128 (43.1%)	1438 (34.4%)

* Denotes statistical significance at *P* < 0.05.

a. Where recorded as ‘yes’ these are reported, and a higher proportion of propranolol deaths had status as ‘not known’ (38.0%) versus where no propranolol involved 31.3%.

b. Number of people with any recorded mental health condition, not sum of all diagnoses individually.

### Propranolol detected by toxicology

Quantifications of propranolol blood levels were available for 168 of the 297 deaths where propranolol was detected by toxicology. The median level was 0.37 mg/L when propranolol was not implicated in causing death and 5.99 mg/L when it was implicated. Among those for whom propranolol was implicated, the median level was higher (*P* = 0.0219) in women (7.20 mg/L) than men (5.01 mg/L).

### Diagnosis and medications where propranolol was involved

Where propranolol was involved in causing death, people were more likely to have a diagnosis of depression (39.1% *v*. 27.1%) or anxiety (22.2% *v*. 8.6%) but less likely to have been known to use drugs (14.8% *v*. 25.2%) compared with suicides where propranolol was not involved (Table 1). Alcohol was co-implicated in 21.9% of deaths (*n* = 65). A median of 5 (IQR 3–7) drugs were concomitantly detected at post-mortem where propranolol was involved in death.

An antidepressant was concomitantly detected at post-mortem in 81.8% (*n* = 243) of suicides involving propranolol, thus was the most commonly co-detected and co-implicated drug class (*n* = 130, 43.8%) (Table 2). Of the antidepressants, selective serotonin reuptake inhibitors (SSRIs) were detected at post-mortem in 51.5% (*n* = 153) of suicides and implicated in 22.2% (*n* = 66). SSRIs were more likely to be co-implicated when propranolol was implicated rather than when propranolol was detected at post-mortem but not implicated (27.2% *v*. 14.2%; *P* = 0.009). When SSRIs were examined individually, this pattern only held true for citalopram, which was the most implicated SSRI (propranolol implicated *n* = 31/184; 16.8% *v*. propranolol at post-mortem *n* = 5/113; 4.4%). Citalopram was implicated more than twice as often as the next most implicated SSRI, fluoxetine (5.7%), and was the most commonly detected SSRI at post-mortem (*n* = 73, 24.6%). Tricyclic antidepressants (TCAs) were detected at post-mortem in 21.2% of deaths (*n* = 63) and were more likely to be detected at post-mortem when propranolol was detected at post-mortem but not implicated, compared with when implicated (27.4% *v*. 17.4%).

**Table 2:** Other drugs at post-mortem or implicated by propranolol status

	All propranolol deaths (*n* = 297)	Propranolol implicated (*n* = 184)	Propranolol at post-mortem but not implicated (*n* = 113)	All propranolol deaths (*n* = 297)	Propranolol implicated (*n* = 184)	Propranolol at post-mortem but not implicated (*n* = 113)	All propranolol deaths with prescribing data (*n* = 254)^a^
	Drugs/drug groups detected at post-mortem			Drugs/drug groups implicated in death			Prescribed
Any antidepressant	243 (81.8%)	146 (79.3%)	97 (85.8%)	130 (43.8%)	87 (47.3%)	43 (38.1%)	192 (45.6%)
Any serotonin-noradrenaline reuptake inhibitor	47 (15.8%)	28 (15.2%)	19 (16.8%)	29 (9.8%)	19 (10.3%)	10 (8.8%)	31 (12.2%)
Any tricyclic antidepressant	63 (21.2%)	32 (17.4%)*	31 (27.4%)*	39 (13.1%)	22 (12.0%)	17 (15.0%)	38 (15.0%)
Any selective serotonin reuptake inhibitor	153 (51.5%)	99 (53.8%)	54 (47.8%)	66 (22.2%)	50 (27.2%)*	16 (14.2%)*	120 (47.2%)
Citalopram	73 (24.6%)	50 (27.2%)	23 (20.4%)	36 (12.1%)	31 (16.8%)*	5 (4.4%)*	55 (24.6%)
Fluoxetine	42 (14.1%)	26 (14.1%)	16 (14.2%)	17 (5.7%)	11 (6.0%)	6 (5.3%)	32 (12.6%)
Paroxetine	5 (1.7%)	2 (1.1%)	3 (2.7%)	2 (0.7%)	1 (0.5%)	1 (0.9%)	4 (1.6%)
Sertraline	43 (14.5%)	26 (14.1%)	17 (15.0%)	16 (5.4%)	10 (5.4%)	6 (5.3%)	33 (13.0%)
Any opioid	120 (40.4%)	56 (30.4%)*	64 (56.6%)*	90 (30.3%)	44 (23.9%)*	46 (40.7%)*	57 (22.4%)
Any sedative/hypnotic	124 (41.8%)	65 (35.3%)*	59 (52.2%)*	50 (16.8%)	33 (17.9%)	17 (15.0%)	73 (28.7%)
Any non-opioid analgesic	96 (32.3%)	60 (32.6%)	36 (31.9%)	28 (9.4%)	18 (9.8%)	10 (8.8%)	0
Any anti-epileptic	50 (16.8%)	19 (10.3%)*	31 (27.4%)*	24 (8.1%)	12 (6.5%)	12 (10.6%)	35 (13.8%)
Gabapentinoid	37 (12.5%)	15 (8.2%)*	22 (19.5%)*	21 (7.1%)	12 (6.5%)	9 (8.0%)	26 (10.2%)
Any antipsychotic	49 (16.5%)	28 (15.2%)	21 (18.6%)	25 (8.4%)	20 (10.9%)	5 (4.4%)	41 (16.1%)

* *P* < 0.05 statistical significance when propranolol implicated versus propranolol at post-mortem but not implicated.

a. Medication prescribed status (yes/no for each drug) known for this subset only.

After antidepressants, sedative-hypnotics (*n* = 124, 41.8%), a category including benzodiazepines and z-drugs, were the most frequently co-detected drug groups at post-mortem, and opioid analgesics were most frequently implicated (*n* = 90, 30.3%). Opioids were less likely to be co-implicated if propranolol was implicated, compared with when propranolol was detected at post-mortem only (23.9% *v*. 40.7%; *P* = 0.002).

Prescription details were available for 254 of the 297 suicides involving propranolol. Of these, 104 (41.9%) were prescribed propranolol. Antidepressants (*n* = 192, 45.6%) were the most commonly co-prescribed medications.

## Discussion

The overall number of suicides reported to the NPSUM decreased between 2010 and 2021, consistent with national data on all suicides.^17^ During the same time, the number of suicides involving propranolol has remained relatively constant overall, accounting for periods of increase and decrease although there has been an increase since 2015. There has been an increasing proportion of suicides that involve propranolol over time. Compared with all other suicides in the NPSUM, those involving propranolol are more common in females, younger age groups and people with a history of depression or anxiety. Antidepressants were the most commonly co-detected and co-implicated drug group at post-mortem, with SSRIs the most common class and citalopram the most common SSRI.

### Contrasting demographics

A lower proportion of people who died by any suicide included in the NPSUM were male (62.9%) than is observed in national suicide data (74.0%).^17^ This difference is likely because most of the suicides in this data-set are poisoning, which accounts for a greater proportion of suicides in women than men.^18^ The difference is even more prominent in the propranolol subset, where 43.4% of suicides were in men, compared with 56.6% in women. An even higher proportion of the 46 propranolol overdose fatalities reported to the UK National Poisons Information Service (NPIS) 2017–2021 were female (77%), and 57% were under the age of 40.^19^ Similarly, 69–80% (2018–2021) of emergency department presentations for propranolol poisoning at the Royal Infirmary of Edinburgh (RIE) were in women with an average age of 30.^20^ The gender disparity could be explained by ease of access to propranolol because propranolol is more commonly prescribed in women than men,^4^ and rates of anxiety are higher in women than men, with increasing incidence over time for women under 55.^21^ However, in the current study, propranolol was prescribed in less than half of people whose suicide involved propranolol and for whom prescribing data are available; and there was no difference in likelihood of implication based on prescribing status. Between 2017 and 2018, of the 339 exposures to propranolol reported to NPIS, at least 43% were known to be the patient's own medicine.^22^ Propranolol is a prescription-only medicine in the UK, so questions about the sources of propranolol must be raised and might include internet purchase, illicit markets and borrowed medication prescribed to someone else. Consistent with the observed findings of an increasing proportion of suicides involving propranolol over time, the number of people presenting with propranolol overdose (90% recorded as intentional self-harm) to the RIE almost doubled between 2018 (*n* = 84) and 2021 (*n* = 160), with the proportion requiring critical care admission increasing from 3.6 to 10%.^20^

### Toxicity of propranolol in overdose

Cardiac effects including bradycardia, hypotension and heart failure are observed in beta-blocker overdose. Propranolol overdose specifically can cause ventricular tachyarrhythmia due to QRS prolongation (ventricular depolarisation), seizures and coma.^1^ Cardiac arrest occurs in most fatal propranolol overdoses.^19^ In a study of beta-blocker poisoning in people in an Iranian hospital, 84.4% of the 255 poisonings involved propranolol alone or in combination.^23^ A Finnish study compared propranolol and metoprolol at post-mortem between 2016–2018.^24^ Although metoprolol (*n* = 416) was detected in more cases than propranolol (*n* = 179), deaths involving propranolol were more often intentional self-poisoning, and benzodiazepines, antidepressants and antipsychotics were more often co-detected.

There is acknowledgement of medicines used in combination with propranolol in poisoning. The aforementioned UK NPIS data detected co-ingestion of antidepressants in almost half of propranolol fatalities.^19^ The HSIB report highlighted concerns about concomitant use of propranolol alongside antidepressants and how the two may interact in overdose.^7^ National guidance in England recommends caution in prescribing TCAs to people at risk of overdose,^25^ due to the high toxicity of TCAs.^26^ However, one-fifth of suicides involving propranolol in this study concomitantly involved TCAs.

### Concomitant involvement of SSRIs, particularly citalopram

There is some evidence of increasing concomitant prescribing of propranolol and SSRIs.^5^ Given that propranolol can be prescribed for the physical symptoms of anxiety, it follows that other medications for the use of anxiety – namely antidepressants, particularly SSRIs, and sedative-hypnotics – were observed in this study population. In this study, SSRIs were more likely to be implicated when propranolol was implicated, rather than when propranolol was detected at post-mortem but not implicated. Implication is determined by coroners’ understanding, informed by toxicology, and therefore it could be that coroners presume that death is because of a combination of the two medications. If this was the case, the same pattern would be observable across all SSRIs. However, this effect was only seen in citalopram and not in the other SSRIs. This may indicate that citalopram and propranolol are more toxic in combination than other SSRI-propranolol combinations. Tentatively, it could be an early signal of a clinically concerning unreported drug–drug interaction.

Two plausible mechanisms include: (1) cardiac rhythm disorders due to the prolongation of the cardiac action potential duration (e.g. via slowing of conduction velocity or repolarisation delay);^1,27,28^ and (2) lowering of the seizure threshold which has been observed in overdose with citalopram^29^ and propranolol^28^ separately. The two postulated mechanisms may be linked, given that seizure risk associated with propranolol overdose has been associated with QRS prolongation.^28^

Citalopram has been shown to be more than three times more toxic in overdose than other SSRIs.^26^ Between 2015 and 2019, citalopram was the most commonly prescribed antidepressant (71 million items), although sertraline prescribing increased by 2 million prescriptions annually, whereas all other antidepressants prescribing rates remained constant.^30^ Citalopram is the most commonly prescribed antidepressant for the treatment of anxiety.^5^

### Suicidal behaviour and propranolol

Suicide, self-harm and suicidal thinking are complex and often multifaceted rather than attributable to a single cause.^31^ However, in the context of understanding suicides involving propranolol, it is important to consider the therapeutic indication and any known adverse effects of propranolol. Depression and anxiety were the most recorded mental health problems among people whose suicide involved propranolol. These conditions are associated with an over five-fold increased risk of self-harm compared with people without the condition.^32^ Depression is listed as a very common side-effect of propranolol^1^ but suicidal behaviour is not. The UK spontaneous reporting system for potential adverse drug reactions (ADRs) includes 37 reports of suicidal and self-injurious behaviour between 2010 and 2021 in people taking propranolol.^33^ These ADR reports indicate that the person was taking the named medication at the time of the reported behaviour but do not indicate causation. In co-authors’ systematic review of observational studies exploring non-psychoactive medicines’ links with suicide and attempted suicide, beta-blockers were not associated with any increased risk.^34^ A cohort study of 1.4 million individuals in Sweden has reported an 8% increase in suicidal behaviour associated with beta-blockers, but this was inconsistent across sensitivity analyses.^35^

### Strengths and limitations

This is the first use of the NPSUM in this time period to explore suicide and the first study in a UK data-set to specifically focus on propranolol-related suicides. The NPSUM contains toxicology data and details of drugs at post-mortem and implicated beyond those normally reported in ONS data in England on both suicides and drug-related deaths.

There are, however, several limitations to this study. This is a descriptive study, thus reports associations not causal inferences. By definition the NPSUM includes reports on deaths related to psychoactive substances. As described earlier, whether propranolol meets this definition is open to interpretation by coroners and their officers, and therefore not all suicides involving propranolol that occurred in England, Wales and Northern Ireland will have been captured in this data-set. The NPSUM is voluntarily reported to by over 80% of coroners, and it is not known whether those who do not report are systematically different from those who report. However, not all deaths are referred to a coroner, and all referred deaths are not subject to toxicological investigations, so even with a 100% coronial reporting rate this would still only provide a representative portion of the true population. Some variables are incompletely recorded in the NPSUM, for example ethnicity, which precluded subgroup comparisons.

### Implications for clinical practice and policy

There was a record of propranolol prescribing in less than half of those for whom propranolol was involved in death. This suggests medication might be borrowed, sourced from private prescribers or illicitly sourced. This is a reminder for healthcare professionals to discuss access to non-prescribed medication when taking a medication history and undertaking medicines reconciliation activities. Limiting access to means of suicide is a key pillar in suicide prevention as described by the World Health Organization.^36^ It is possible that people are using propranolol in overdose due to ease of access in the community, which could be perpetuated by possible patient and clinician views of relative safety versus alternatives.^5^ The co-prescription of propranolol with antidepressants, particularly citalopram, may need to be considered while balancing the clinical needs for appropriate treatment against likelihood of self-poisoning. However, more evidence is needed to understand whether prescribing should be channelled away from citalopram and towards other SSRIs, when in combination with propranolol.

### Future research priorities

An understanding of any adverse clinical outcomes, including suicide but also beyond (e.g. seizure, sudden cardiac death) when citalopram and propranolol are used in combination, requires investigation. Future work by our team will investigate this through robustly designed epidemiological studies using routinely collected healthcare data. A better understanding of where medication involved in self-poisonings are sourced from is required, not just related to propranolol but across medicine poisonings. This might support recommendations and policy implementations for medicine access, beyond what is possible within the auspices of prescribing, if this was not the source of the medication.

## Conclusions

A small number, but increasing proportion, of suicides reported to the NPSUM involve propranolol. Suicides involving propranolol occur more in females and younger ages than all suicides in the NPSUM. Given that there have been substantial increases in the prescribing of propranolol in recent years, and that the combination of propranolol and antidepressants is commonly seen for the management of anxiety with or without depression, vigilance to the combined toxicity profile of drugs concomitantly prescribed with propranolol may be pertinent.

## Supporting information

Gorton et al. supplementary material Gorton et al. supplementary material

## Data Availability

The data and analytic code that support the findings of this study are available on request from the corresponding author, H.C.G. The data are not publicly available because they contain information that could compromise the privacy of research participants. The analytic code is not publicly available because there is not a corresponding publicly available data-set to which the code is applicable.
